# The prognostic and predictive role of the neutrophil-to-lymphocyte ratio and the monocyte-to-lymphocyte ratio in early breast cancer, especially in the HER2+ subtype

**DOI:** 10.1007/s10549-020-05925-7

**Published:** 2020-09-18

**Authors:** Satu Tiainen, Kirsi Rilla, Kirsi Hämäläinen, Sanna Oikari, Päivi Auvinen

**Affiliations:** 1 Cancer Center, Kuopio University Hospital, P.O. Box 100, 70029 Kuopio, Finland; 2 Institute of Clinical Medicine, University of Eastern Finland, P.O. Box 1627, 70211 Kuopio, Finland; 3 Institute of Biomedicine, University of Eastern Finland, P.O. Box 1627, 70211 Kuopio, Finland; 4 Imaging Center, Clinical Pathology, Kuopio University Hospital, P.O. Box 100, 70029 Kuopio, Finland; 5 Institute of Clinical Medicine, Clinical Pathology and Forensic Medicine, University of Eastern Finland, P.O. Box 1627, 70211 Kuopio, Finland; 6 Biocenter Kuopio and Cancer Center of Eastern Finland, University of Eastern Finland, P.O. Box 1627, 70211 Kuopio, Finland

**Keywords:** Breast cancer, NLR, MLR, Trastuzumab, HER2

## Abstract

**Purpose:**

The aim of this study was to investigate the prognostic impact of two systemic inflammatory markers, the neutrophil-to-lymphocyte ratio (NLR) and the monocyte-to-lymphocyte ratio (MLR), and their possible predictive role regarding the efficacy of adjuvant trastuzumab, in 209 early breast cancer cases, 107 of which were HER2-positive.

**Methods:**

Baseline NLR and MLR values were divided into two groups, high and low, according to cut-off-points determined from the ROC curve (2.2 for NLR and 0.22 for MLR). Cox’s model was utilized for survival analyses.

**Results:**

High NLR and MLR correlated with poor overall survival (OS) and breast cancer specific survival (BCSS) among all the patients (*p* ≤ 0.030). Among the HER2+ patients whose adjuvant treatment did not include trastuzumab (*n* = 64), the survival rates were remarkably lower in patients with a high NLR as compared to those with low; 31% vs. 71% for OS and 42% vs. 74% for BCSS (*p* ≤ 0.014). Similarly, high MLR correlated with poor survival among these patients (*p* ≤ 0.020). On the contrary, among the patients who had received adjuvant trastuzumab (*n* = 43), NLR or MLR did not correlate with survival. Furthermore, trastuzumab was beneficial for the HER2+ patients with high NLR/MLR, while the survival of the HER2+ patients with low NLR/MLR was good irrespective if they received adjuvant trastuzumab.

**Conclusions:**

Our results suggest that trastuzumab modulates the systemic inflammatory conditions and overcomes the poor prognostic impact of high NLR/MLR. This finding may also provide a rationale for combining trastuzumab with immuno-oncological treatments in HER2+ breast cancer.

**Electronic supplementary material:**

The online version of this article (10.1007/s10549-020-05925-7) contains supplementary material, which is available to authorized users.

## Introduction

Systemic inflammatory state of the host and the inflammatory response in the tumor microenvironment have a remarkable impact on cancer progression [1, 2]. Neutrophils secrete cytokines that promote cancer growth [3], and they may also suppress the immune responses mediated by lymphocytes [4]. Furthermore, monocytes are able to suppress lymphocyte activation and enhance tumor progression [5]. On the other hand, lymphocytes have an important role in the immune responses against cancer both in the circulation and in the tumor microenvironment, e.g., via T-cell mediated cellular cytotoxicity [6]. Therefore, the blood neutrophil-to-lymphocyte ratio (NLR) and monocyte-to-lymphocyte ratio (MLR) can be considered as indicators of the host’s systemic inflammatory state [7], i.e., high NLR and MLR may reflect poor anti-tumor immunity.

Several studies have investigated the prognostic role of NLR in breast cancer (BC) [7]. In two rather recent meta-analyses, a high NLR was shown to predict poor overall survival (OS) and disease free survival (DFS) among BC patients [8, 9]. The associations were evident especially among those patients with hormone receptor (HR) negative and/or human epidermal growth factor receptor 2 (HER2) negative BC but less clear among the HER2+ patients [8, 9]. The impact of MLR on BC prognosis has been less extensively studied. Some publications suggest that a high MLR associates with a poor outcome in BC [7, 10] but there are also studies reporting no correlation between MLR and BC outcome [7, 11].

One of the major advances in the treatment of BC has been the development of trastuzumab, a HER2 antibody, which has remarkably improved the outcome of HER2+ patients [12]. A part of trastuzumab’s anti-tumor effects are mediated via the immune system, especially through antibody-dependent cellular cytotoxicity (ADCC) [6]. First, trastuzumab binds to the surface of cancer cells and activates the innate immune response, resulting in the death of cancer cells by macrophages and natural killer (NK) cells. Dying cancer cells release trastuzumab-coated antigens, which activate cytotoxic T-lymphocytes leading to an adaptive immune response and death of the cancer cells presenting this antigen [6]. Indeed, high levels of tumor infiltrating lymphocytes (TILs) have been shown to associate with a better response to trastuzumab in HER2+ BC [13]. There are only a few studies investigating the possible predictive role of the blood inflammatory cells in HER2+ BC treated with trastuzumab, and the results have been conflicting [14–16].

The aim of this study was to investigate the prognostic impact of both NLR and MLR in a material of 209 BC patients, 107 of them HER2+. Patients were diagnosed between 2001 and 2008 and 40% of the HER2+ patients had received adjuvant trastuzumab, since adjuvant trastuzumab only became widely available in Finland in 2005. Thus, this material provides a rather unique opportunity to investigate also if these two markers of systemic inflammation, NLR and MLR, have a predictive role regarding the efficacy of adjuvant trastuzumab treatment.

## Material and methods

### Patient material

The primary patient material included 278 BC cases diagnosed in Kuopio University Hospital between 2001–2008; first all HER2+ operated cases with adequate tissue blocks (*n* = 139) were included and then the same number of HER2− cases matched for the time of operation and age were selected [17]. The information of the complete blood count with white blood cell (WBC) differential was available for 209 eligible patients (107 HER2+ and 102 HER2−), which is the number of patients included in the present study. Clinicopathological data including the information of adjuvant treatments, laboratory tests and survival were collected manually from the patient records of Kuopio University Hospital. The conduction of this study was in accordance with the Declaration of Helsinki, and the ethical approval was provided by the Ethics Committee of the University of Eastern Finland (February 24, 2009, 19//2009).

### Laboratory tests

The complete blood count with WBC differential was determined from the blood samples in the laboratory of Kuopio University Hospital with an Advia 120 Hematology System (Bayer Diagnostics Co., Tarrytown, NY, USA). Blood samples taken within 3 months after BC surgery and before the initiation of adjuvant treatments were included but blood samples taken during an infection were excluded. The ratios between the WBC counts were calculated as follows; NLR = neutrophils divided by lymphocytes and MLR = monocytes divided by lymphocytes. In the statistical analyses, the NLR and MLR values were each graded as low or high according to optimal cut-off-points determined from the receiver operating characteristic (ROC) curve.

### Immunohistochemistry and in situ hybridization

HER2 expression (chromogenic in situ hybridization test) and other standard histopathological parameters, e.g., tumor size, nodal status, grade, histological type and HR status (immunohistochemistry) were determined according to international guidelines [18] in Kuopio University Hospital, Department of Pathology at the time of diagnosis. The cut-off points for estrogen receptor (ER) and progesterone receptor (PR) positivity were 10% according to the guidelines at the time of diagnosis, and cases were defined as HR+ when either ER or PR was positive.

### Statistical analyses

IBM SPSS Statistics version 25 (IBM Corporation, Armonk, NY, USA) was utilized for the statistical analyses. The optimal cut-off points for NLR and MLR were determined from the ROC curve. Chi-square test was used for calculating the differences between the investigated factors, Cox’s model for survival analyses and the Kaplan Meier method for plotting the survival curves. OS and BC specific survival (BCSS) were determined as the time from diagnosis to the date of death or end of follow-up; for OS death from any cause was counted as an event, and death from BC for BCSS. The survival rates were calculated at the end of the follow-up time. In the Cox’s multivariate survival analyses the factors included were NLR, MLR, tumor size (T2-4 vs. T1), nodal status (N1-3 vs N0), HR and HER2 status, and for HER2+ subgroup also adjuvant trastuzumab. *p*-values ≤ 0.05 were regarded as statistically significant.

## Results

The WBC differential was available for 209 eligible patients (107 HER2+ and 102 HER2−) and the demographics of these cases are presented in Table 1. The median follow-up time was 10.4 years (range 0.5–15.2). Median age was 58.1 years (range 32.3–82.7) and 35% of the patients were premenopausal (Table 1). Deaths and breast cancer relapses occurred more often among the HER2+ patients than among the HER2− patients (*p* ≤ 0.006) (Table 1).

**Table 1:** Demographics of the cases

	All (*n* = 209)	HER2+ (*n* = 107)	HER2− (*n* = 102)
	*n* (%)	*n* (%)	*n* (%)
T1	124 (59)	57 (53)	67 (66)
T2	70 (34)	42 (39)	28 (27)
T3	5 (2)	3 (3)	2 (2)
T4	10 (5)	5 (5)	5 (5)
N0	78 (37)	35 (33)	43 (42)
N1	96 (46)	46 (43)	50 (49)
N2	25 (12)	17 (16)	8 (8)
N3	10 (5)	9 (8)	1 (1)
Grade 1	20 (9)	4 (4)	16 (16)
Grade 2	95 (46)	38 (35)	57 (56)
Grade 3	94 (45)	65 (61)	29 (28)
Ductal	170 (81)	91 (85)	79 (77)
Lobular	22 (11)	8 (7.5)	14 (14)
Other	17 (8)	8 (7.5)	9 (9)
HR positive	150 (72)	62 (58)	88 (86)
HR negative	59 (28)	45 (42)	14 (14)
Premenopausal	74 (35)	37 (35)	37 (36)
Postmenopausal	135 (65)	70 (65)	65 (64)
Any death	57 (27)	40 (37)	17 (17)
Death due to BC	42 (20)	34 (32)	8 (8)
BC any relapse	64 (31)	42 (39)	22 (22)
BC distant relapse	50 (24)	36 (34)	14 (14)
NLR low	136 (65)	66 (62)	70 (69)
NLR high	73 (35)	41 (38)	32 (31)
MLR low	120 (57)	62 (58)	58 (57)
MLR high	89 (43)	45 (42)	44 (43)

*T* tumor classification, *N* nodal classification, *HR* hormone receptor, *BC* breast cancer, *NLR* neutrophil-to-lymphocyte ratio, *MLR* monocyte-to-lymphocyte ratio

Median time from the BC surgery to the blood sample collection was 6.0 weeks (range 0.3–12.8). The optimal cut-off-points calculated with the ROC curve were 2.2 for NLR (AUC 0.624, 95% CI 0.54–0.71, *p* = 0.006) and 0.22 for MLR (AUC 0.597, 95% CI 0.51–0.68, *p* = 0.031). High NLR (≥ 2.2) was found in 35% and high MLR (≥ 0.22) in 43% of the patients (Table 1). In the HER2+ subgroup, a high NLR was found in 38% and a high MLR in 42% of the patients; the corresponding values in the HER2− subgroup were 31% for high NLR and 43% for high MLR (Table 1). Similarly, in the HR+ subgroup (*n* = 150), a high NLR was found in 35% and a high MLR in 41% of the patients, and the corresponding values in the HR− subgroup (*n* = 59) were 34% for high NLR and 46% for high MLR. There were no correlations between NLR or MLR and standard prognostic factors, i.e., tumor size, nodal status, grade, HER2 or HR status (data not shown).

### High NLR and MLR correlate with poor OS and BCSS

Among all the patients (*n* = 209), the OS rate at the end of the follow-up was worse in patients with a high NLR as compared to those with a low NLR, 60% vs. 79% (*p* = 0.004), and similarly the BCSS rates were 71% vs. 85% (*p* = 0.023) for high and low NLR, respectively (Table 2). A high MLR also correlated with poor survival as the OS rate was 61% vs. 82% (*p* = 0.001) and the BCSS rate 73% vs. 85% (*p* = 0.030) for high and low MLR, respectively (Table 2). In the HR+ subgroup (*n* = 150), high NLR and MLR correlated with poor OS (*p* ≤ 0.044), as well as in the HR− subgroup (*n* = 59) (*p* ≤ 0.044) (Table 2). The distributions of the standard prognostic histopathological parameters were similar in NLR/MLR high and low groups among all the patients and in the HR+ and HR− subgroups (data not shown). There were only 14 patients with triple-negative BC and no correlations were found between NLR or MLR and survival (Table 2).

**Table 2:** OS and BCSS rates at the end of the follow-up according to NLR and MLR values

	OS	*p* value	HR	95% CI	BCSS	*p* value	HR	95% CI
All (*n* = 209)								
NLR high	60%	0.004	2.13	1.27–3.58	71%	0.023	2.02	1.10–3.70
NLR low	79%				85%			
MLR high	61%	0.001	2.41	1.41–4.12	73%	0.030	1.97	1.07–3.64
MLR low	82%				85%			
HER2+ (*n* = 107)								
NLR high	46%	0.011	2.25	1.21–4.21	59%	0.084	1.81	0.92–3.55
NLR low	73%				74%			
MLR high	47%	0.005	2.46	1.30–4.63	58%	0.040	2.04	1.04–4.01
MLR low	74%				76%			
HER2− (*n* = 102)								
NLR high	78%	0.393	1.52	0.58–4.01	88%	0.293	2.11	0.53–8.42
NLR low	86%				94%			
MLR high	75%	0.053	2.68	0.99–7.28	89%	0.232	2.40	0.57–10.06
MLR low	90%				95%			
HR+ (*n* = 150)								
NLR high	70%	0.044	2.06	1.02–4.18	79%	0.059	2.33	0.97–5.64
NLR low	85%				91%			
MLR high	69%	0.014	2.47	1.20–5.10	82%	0.157	1.89	0.78–4.57
MLR low	86%				90%			
HR− (*n* = 59)								
NLR high	35%	0.023	2.44	1.13–5.28	50%	0.118	1.96	0.84–4.53
NLR low	67%				69%			
MLR high	41%	0.044	2.25	1.02–4.97	52%	0.120	1.96	0.84–4.59
MLR low	69%				72%			
TNBC (*n* = 14)								
NLR high	50%	0.294	2.86	0.40–20.35	75%	0.758	1.46	0.13–16.15
NLR low	80%				80%			
MLR high	67%	0.876	1.17	0.16–8.32	83%	0.655	0.58	0.05–6.40
MLR low	75%				75%			

*OS* overall survival, *BCSS* breast cancer specific survival, *NLR* neutrophil-to-lymphocyte ratio, *MLR* monocyte-to-lymphocyte ratio, *HR* hazard ratio, *CI* confidence interval, *HR+* hormone receptor positive, *TNBC* triple-negative breast cancer, *HR−* hormone receptor negative

Among the HER2+ patients (*n* = 107), OS was inferior in patients with a high NLR as compared to those with a low NLR, 46% vs. 73% (*p* = 0.011), and BCSS showed a similar trend, 59% vs. 74%, respectively (*p* = 0.084) (Table 2). Similarly, the OS and BCSS rates were 47% vs. 74% (*p* = 0.005) and 58% vs. 76% (*p* = 0.040) for patients with high and low MLR, respectively (Table 2). In addition, 41.5% (17/41) and 44% (20/45) of the patients with high NLR or MLR, respectively, suffered a distant relapse during the follow-up as compared to the corresponding values of 29% (19/66) of patients with low NLR and 26% (16/62) with low MLR (*p* = 0.177 and *p* = 0.044, respectively). Among the HER2− patients (*n* = 102) there were no statistically significant differences in OS, BCSS (Table 2) or recurrence rates (data not shown) according to either NLR or MLR. There were no significant differences in the distributions of the standard histopathological factors in NLR/MLR high and low groups among the HER2+ or HER2− patients (data not shown).

### High NLR and MLR correlate with poor survival among HER2+ patients only if not treated with adjuvant trastuzumab

Of the HER2+ patients, 82% had received chemotherapy, 53% hormonal therapy and 95% radiation therapy among adjuvant treatments (Table 3). Also, 40% (43/107) had received adjuvant trastuzumab but 60% (64/107) had not since they were treated before adjuvant trastuzumab was included in the national and international guidelines (Table 3). All the patients treated with adjuvant trastuzumab received also chemotherapy, of them 93% (40/43) anthracycline and 81% (35/43) taxane. Of the HER2+ patients who did not receive trastuzumab, chemotherapy was administered to 70% (45/64) and the regimen included anthracycline in 44% (28/64) and taxane in only 8% (5/64) of the cases. Otherwise the adjuvant treatments and the standard histopathological factors distributed similarly (data not shown). Of the patients treated with trastuzumab, 74% (32/43) had received a short duration adjuvant trastuzumab, i.e., three doses together with chemotherapy, while 26% (11/43) had received trastuzumab for 1 year.

**Table 3:** Adjuvant treatments of the HER2+ patients

	HER2+ (*n* = 107)
	*n* (%)
Any chemo	88 (82)
Anthracycline	68 (64)
Taxane	40 (37)
Any trastuzumab	43 (40)
Trastuzumab with taxane	30 (28)
Trastuzumab with vinorelbine	6 (6)
Trastuzumab after chemo	7 (6.5)
Hormonal therapy	57 (53)
Radiation therapy	102 (95)

*Chemo* chemotherapy

**Fig. 1:**
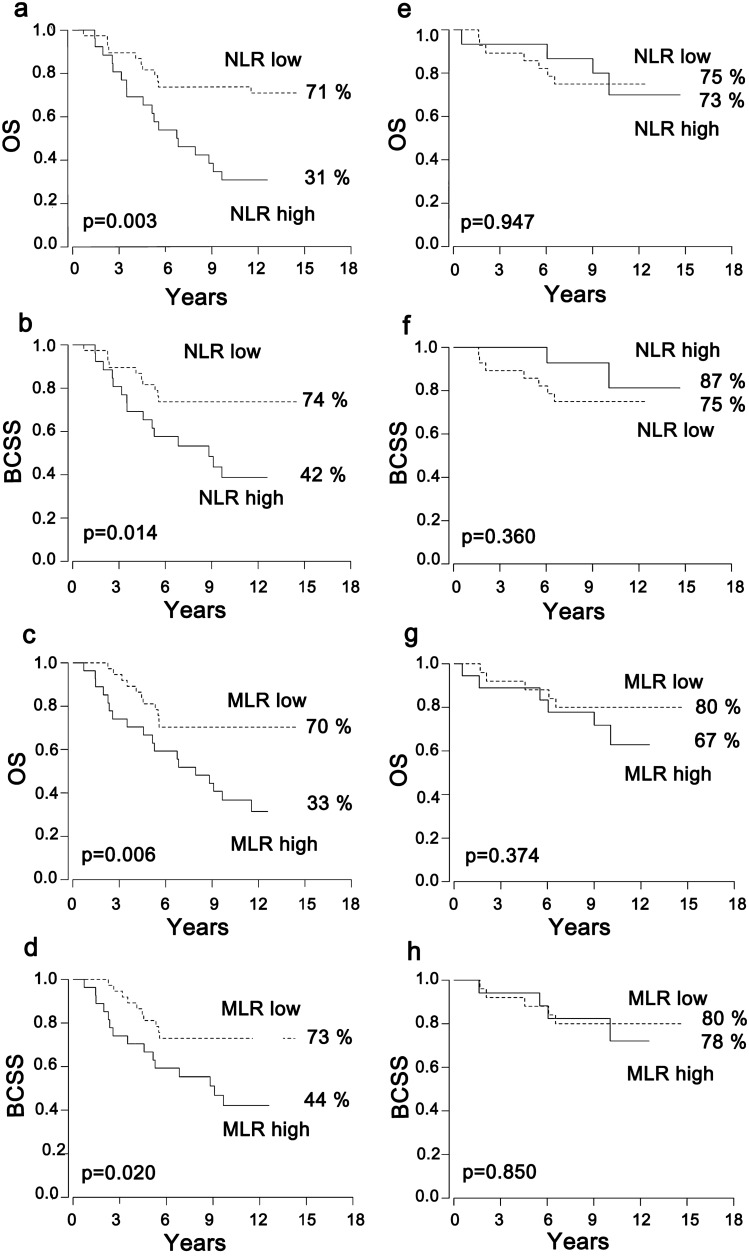
High NLR and MLR correlate with poor survival among HER2+ patients only if their treatment does not include adjuvant trastuzumab. Overall survival (OS) and breast cancer specific survival (BCSS) curves of the HER2+ patients treated without adjuvant trastuzumab subdivided according to the neutrophil-to-lymphocyte ratio (NLR) (panels **a** and **b**) and the monocyte-to-lymphocyte ratio (MLR) (panels **c** and **d**), and among patients treated with adjuvant trastuzumab subdivided according to the NLR in panels **e**–**f** and MLR in panels **g**–**h**

Among the HER2+ patients whose adjuvant treatment did not include trastuzumab, the outcome of the patients with a high NLR was poor. As presented in Fig. 1 and Table 4, the OS rate at the end of the follow-up was only 31% among the patients with a high NLR as compared to 71% among the patients with a low NLR (*p* = 0.003) (Fig. 1a, Table 4). In addition, the BCSS rate was clearly lower in patients with a high NLR as compared to those with a low NLR, 42% vs. 74% (*p* = 0.014), respectively (Fig. 1b, Table 4). Similarly, a high MLR correlated with poor OS and BCSS among these patients (*p* ≤ 0.020) (Fig. 1c, d, Table 4). Furthermore, distant relapses were more frequent among the patients with either high NLR or MLR in comparison to those with low values, 58% (15/26) vs. 32% (12/38) (*p* = 0.038) for NLR, and 59% (16/27) vs. 30% (11/37) (*p* = 0.018) for MLR. The standard histopathological factors distributed similarly in the NLR/MLR high and low groups (Supplementary Table S1). Surprisingly, among the HER2+ patients who had received adjuvant trastuzumab, there were no differences in OS or BCSS according to either NLR or MLR (Fig. 1e–h, Table 4). The trastuzumab treated groups were otherwise in balance, but the frequency of T1 tumors was higher in the NLR high group than in the NLR low group (*p* = 0.011) (Supplementary Table S1).

**Table 4:** Survival analyses of the HER2+ patients treated without or with adjuvant trastuzumab

	OS	*p* value	HR	95% CI	BCSS	*p* value	HR	95% CI
HER2+ no adj trastuzumab *n* = 64								
NLR high	31%	0.003	3.16	1.49–6.73	42%	0.014	2.78	1.24–6.21
NLR low	71%				74%			
MLR high	33%	0.006	2.95	1.39–6.27	44%	0.020	2.64	1.19–5.90
MLR low	70%				73%			
HER2+ adj trastuzumab *n* = 43								
NLR high	73%	0.947	0.96	0.28–3.29	87%	0.360	0.48	0.10–2.31
NLR low	75%				75%			
MLR high	67%	0.374	1.72	0.52–5.63	78%	0.850	1.14	0.30–4.24
MLR low	80%				80%			
HER2+ NLR high *n* = 41								
Adj trastuzumab	73%	0.017	0.27	0.09–0.79	87%	0.017	0.16	0.04–0.72
No adj trastuzumab	31%				42%			
HER2+ NLR low *n* = 66								
Adj trastuzumab	75%	0.859	0.92	0.35–2.40	75%	0.873	0.92	0.35–2.43
No adj trastuzumab	71%				74%			
HER2+ MLR high *n* = 45								
Adj trastuzumab	67%	0.064	0.41	0.16–1.05	78%	0.044	0.32	0.11–0.97
No adj trastuzumab	33%				44%			
HER2+ MLR low *n* = 62								
Adj trastuzumab	80%	0.391	0.63	0.22–1.81	80%	0.512	0.70	0.24–2.04
No adj trastuzumab	70%				73%			

*Adj* adjuvant, *OS* overall survival, *HR* hazard ratio, *CI* confidence interval, *BCSS* breast cancer specific survival, *NLR* neutrophil-to-lymphocyte ratio, *MLR* monocyte-to-lymphocyte ratio

When analyzed contrariwise, the OS and BCSS rates were similar among the HER2+ patients with a low NLR irrespective if they had received adjuvant trastuzumab (Supplementary Fig. S1e-f, Table 4). On the contrary, among the patients with a high NLR, the OS was 73% in patients with adjuvant trastuzumab vs. 31% in those not receiving this therapy (*p* = 0.017) (Supplementary Fig. S1a, Table 4), and the corresponding BCSS rates were 87% vs. 42% (*p* = 0.017) (Supplementary Fig. S1b, Table 4). Thus, adjuvant trastuzumab conferred a survival advantage especially among those HER2+ patients with a high NLR at baseline. Similarly, among the HER2+ patients with a high MLR, BCSS was prolonged (*p* = 0.044) and there was also a trend towards improved OS (*p* = 0.064) among those who received adjuvant trastuzumab (Supplementary Fig. S1c-d, Table 4), whereas among the patients with a low MLR, there were no differences in survival irrespective whether or not they received adjuvant trastuzumab (Supplementary Fig. S1g-h, Table 4).

### Cox multivariate analyses

In the Cox multivariate analyses, the significant prognostic factors for OS among the HER2+ patients were HR status and adjuvant trastuzumab (*p* ≤ 0.043); for BCSS these were nodal status, HR status and adjuvant trastuzumab (*p* ≤ 0.041) (Table 5). Among the patients treated with adjuvant trastuzumab (*n* = 43), none of the factors reached statistical significance (data not shown). Among the HER2+ patients treated without adjuvant trastuzumab (*n* = 64), HR status (*p* = 0.013, HR 0.38, 95% CI 0.18–0.82) and NLR (*p* = 0.046, HR 3.11, 95% CI 1.02–9.50) were significant prognostic factors for OS, and HR status also for BCSS (*p* = 0.023, HR 0.39, 95% CI 0.17–0.88). Table 5 shows the results of the COX multivariate survival analyses also among all the patients and in the HR+, HR− and HER2− subgroups.

**Table 5:** COX multivariate analyses for OS and BCSS

OS All (*n* = 209)	*p* value	HR	95% CI	BCSS all	*p* value	HR	95% CI
NLR	0.069	1.75	0.96–3.21	NLR	0.063	1.97	0.97–4.01
MLR	0.055	1.83	0.99–3.41	MLR	0.424	1.34	0.65–2.74
Tumor size	0.008*	2.11	1.22–3.65	Tumor size	0.006*	2.54	1.31–4.90
Nodal status	0.030*	2.12	1.07–4.19	Nodal status	0.011*	3.43	1.32–8.88
HR status	0.009*	0.48	0.28–0.83	HR status	0.008*	0.42	0.23–0.80
HER2 status	0.026*	1.96	1.08–3.55	HER2 status	0.004*	3.20	1.44–7.07

OS HER2+ (*n* = 107)	*p* value	HR	95% CI	BCSS HER2+	*p* value	HR	95% CI
NLR	0.070	2.09	0.94–4.61	NLR	0.168	1.83	0.78–4.32
MLR	0.268	1.56	0.71–3.45	MLR	0.489	1.35	0.58–3.17
Tumor size	0.117	1.69	0.88–3.24	Tumor size	0.092	1.85	0.91–3.78
Nodal status	0.066	2.22	0.95–5.19	Nodal status	0.023*	3.46	1.19–10.09
HR status	0.013*	0.45	0.24–0.84	HR status	0.022*	0.45	0.22–0.89
Adj trastuzumab	0.043*	0.48	0.24–0.98	Adj trastuzumab	0.041*	0.45	0.21–0.97

OS HER2− (*n* = 102)	*p* value	HR	95% CI	BCSS HER2−	*p* value	HR	95% CI
NLR	0.892	0.93	0.33–2.64	NLR	0.799	1.22	0.26–5.66
MLR	0.053	2.92	0.99–8.61	MLR	0.279	2.47	0.48–12.70
Tumor size	0.008*	4.06	1.44–11.40	Tumor size	0.017*	14.24	1.60–126.59
Nodal status	0.132	2.40	0.77–7.47	Nodal status	0.221	3.82	0.45–32.67
HR status	0.243	0.51	0.16–1.59	HR status	0.151	0.34	0.08–1.49

OS HR+ (*n* = 150)	*p* value	HR	95% CI	BCSS HR+	*p* value	HR	95% CI
NLR	0.569	1.28	0.54–3.04	NLR	0.272	1.91	0.60–6.08
MLR	0.068	2.27	0.94–5.47	MLR	0.628	1.33	0.42–4.24
Tumor size	0.107	1.84	0.88–3.88	Tumor size	0.132	2.06	0.80–5.30
Nodal status	0.132	1.91	0.82–4.43	Nodal status	0.057	3.47	0.97–12.47
HER2 status	0.047*	2.10	1.01–4.35	HER2 status	0.006*	4.19	1.50–11.66

OS HR− (*n* = 59)	*p* value	HR	95% CI	BCSS HR−	*p* value	HR	95% CI
NLR	0.059	2.30	0.97–5.48	NLR	0.187	1.88	0.74–4.78
MLR	0.401	1.47	0.60–3.59	MLR	0.623	1.27	0.49–3.30
Tumor size	0.034*	2.56	1.07–6.11	Tumor size	0.042*	2.73	1.04–7.20
Nodal status	0.106	2.77	0.81–9.51	Nodal status	0.087	3.66	0.83–16.23
HER2 status	0.203	2.03	0.68–6.02	HER2 status	0.201	2.24	0.65–7.71

*OS* overall survival, *BCSS* breast cancer specific survival, *HR* hazard ratio, *CI* confidence interval, *NLR* neutrophil-to-lymphocyte ratio, *MLR* monocyte-to-lymphocyte ratio, *HR status* hormone receptor status, *Adj* adjuvant, *HR+* hormone receptor positive, *HR−* hormone receptor negative

*p ≤ 0.05

## Discussion

In this study, we found that the outcome of the HER2+ BC patients with a high NLR or MLR was poor if their adjuvant treatment did not include trastuzumab. Trastuzumab was especially beneficial for those HER2+ patients with a high baseline NLR or MLR, while the survival of the HER2+ patients with a low NLR or MLR was good irrespective if they received adjuvant trastuzumab. Both high NLR and MLR correlated with poor survival also in the whole patient material and in HR+ and HR− subgroups.

Based on the results emerging from the present study, we hypothesize that trastuzumab modulates the systemic inflammatory state of the host and overcomes the poor prognostic impact of high baseline NLR and MLR. Indeed, it has been shown in advanced HER2+ BC that after one cycle of trastuzumab emtansine the blood lymphocyte counts were elevated, resulting in a decreased NLR and improved outcome [19]. In another study, after one cycle of trastuzumab, the neutrophil count decreased among patients exhibiting a response to trastuzumab but increased among those who did not benefit from trastuzumab [20]. It is also known that at least some chemotherapeutic agents have immune-modulatory effects, e.g., paclitaxel may sensitize tumor cells to cytotoxic T-lymphocyte mediated cell death [21] which also plays a crucial role in trastuzumab’s mechanism of action. Thus, the synergistic effect of trastuzumab with chemotherapy, especially with taxanes, seems to be at least in part explained by the improvement in the anti-tumor immune response [22]. Since in the present study the patients received trastuzumab in combination with chemotherapy, 81% with taxane, it is not possible to analyse if the possible immune-modulatory effect is due to trastuzumab alone, chemotherapy or both.

The host’s inflammatory state also seems to influence on the efficacy of immuno-oncological (IO) treatments, e.g., programmed death-1/programmed death ligand-1 (PD-1/PD-L1) inhibitors. In recent years, IO-treatments have shown remarkable efficacy in many cancer types but in BC only a small subset of patients obtain long-lasting responses [23]. It is important to understand the mechanisms behind treatment responses to expand the proportion of patients benefiting from these treatments*.* A high NLR has been reported to be a marker of poor prognosis also in cancer patients treated with PD-1/PD-L1 inhibitors [24]. Thus, trastuzumab’s possible ability to modulate the blood lymphocyte and neutrophil counts provides a rationale for combining trastuzumab with IO-treatments. In fact, there are ongoing clinical trials investigating the efficacy of these combination treatment strategies [23]. Furthermore, it would be important to understand the mechanisms behind the increased NLR/MLR as they may reveal new risk factors for poor outcome in BC. In many diseases, such as cancer, infections or chronic inflammatory diseases, a high NLR/MLR might reflect the state of host’s systemic inflammation before the illness, the immunological response mounted against the illness or some aspects of the illness itself. Interestingly, the new COVID-19-infection seems to be especially serious among patients with a high NLR [25] and more often severe also among patients with chronic inflammatory conditions. Here, NLR or MLR did not correlate with the standard prognostic factors, suggesting that in BC NLR and MLR are not a consequence of the aggressiveness or extent of the cancer itself but otherwise modulate tumor progression and treatment responses.

One recent retrospective study has compared the prognostic value of the NLR among HER2+ patients treated with or without adjuvant trastuzumab [16]. In contrast to our results, NLR was not prognostic among HER2+ patients treated without trastuzumab but showed prognostic value among the patients with 1-year adjuvant trastuzumab. The difference in the 3-year DFS rate was rather small, 95.3% vs. 90.5% for low and high NLR groups, respectively, with a rather short median follow-up time of 20 months [16]. In our study, with a long follow-up time (median 10.4 years), the prognostic impact of both NLR and MLR according to OS, BCSS and distant relapse rate was found among the HER2+ patients treated without adjuvant trastuzumab but not among those who did receive trastuzumab. Importantly, NLR remained as a significant prognostic factor for OS also in the COX multivariate analysis. There are several explanations for these contradicting results, e.g., there are differences in determining the optimal cut-off-point for NLR, in the timing of the pre-treatment blood samples, in the treatments received in addition to trastuzumab and in the length of trastuzumab treatment. Furthermore, there is no knowledge of the inflammatory state of the tumor microenvironment, e.g., TILs, tumor associated macrophages (TAMs) and NK-cells, all of which probably exert an influence on the response to trastuzumab [6].

In line with previous studies [9], a high NLR correlated here with poor survival among the HR− patients, but also in the HR+ subgroup. A long follow-up period for survival and BC specific outcomes may be especially important in HR+ BC in which the recurrences often occur quite late. Furthermore, we found a correlation between MLR and survival among the HER2+, HR+ and HR− patients suggesting that also monocytes have an important role in BC progression. Instead, in the HER2− and triple-negative subgroups, we did not find statistically significant correlations between NLR or MLR and survival, but among the HER2− patients the number of events was rather small, and the triple-negative subgroup was too small for reliable statistical analyses.

The strengths of our study include its unique patient material in which approximately half of the HER2+ patients had received adjuvant trastuzumab. Two other strengths are the long follow-up period and reliable patient records. This study has also limitations; first, since this is a retrospective observational study, the causal relationships between the investigated factors remain hypothetical. Second, chemotherapy was administered more often to patients who received adjuvant trastuzumab compared to those who did not, which must be considered in the interpretation of the results since also chemotherapy may modulate inflammatory responses as discussed above. Third, the number of patients in some of the subgroup analyses was small. Thus, the results will need to be confirmed in larger randomized studies.

## Conclusions

This study detected a prognostic impact of both NLR and MLR, i.e., two inexpensive and readily available inflammatory markers, in BC, including the HER2+ subgroup. Our finding that also a high MLR correlates with poor survival strengthens the idea that in addition to neutrophils and lymphocytes, monocytes also play an important role in BC progression and treatment responses*.* Interestingly, the prognostic impact of NLR and MLR was present only among those HER2+ patients who had not received adjuvant trastuzumab, and adjuvant trastuzumab was beneficial especially for those patients with a high NLR or MLR before the initiation of adjuvant treatment. These results suggest that trastuzumab, at least together with chemotherapy, may improve the inflammatory state of the host and overcome the poor prognostic impact of high baseline NLR and MLR, which could also provide a rationale for combining trastuzumab with IO-treatments. More studies are needed to clarify the role of inflammatory cells in the regulation of tumor progression and treatment responses to further improve BC treatments and outcome.

## Electronic supplementary material

Below is the link to the electronic supplementary material.

Supplementary Fig. S1 Adjuvant trastuzumab improves survival especially among the HER2+ patients with high values of NLR or MLR. Overall survival (OS) and breast cancer specific survival (BCSS) curves of the HER2+ patients with a high neutrophil-to-lymphocyte ratio (NLR) treated with or without adjuvant trastuzumab (Adj T) (panels a-b), with a high monocyte-to-lymphocyte ratio (MLR) (panels c-d), with a low NLR (panels e-f) and with a low MLR (panels g-h). (TIF 292980 kb)

Supplementary file2 (DOCX 18 kb)

## Data Availability

The datasets generated and/or analyzed during the current study are not publicly available due to the fact that they contain information that could compromise research participant privacy but may be available from the corresponding author on reasonable request and with required permissions.
