# Supplementary material for: The prognostic and predictive role of the neutrophil-to-lymphocyte ratio and the monocyte-to-lymphocyte ratio in early breast cancer, especially in the HER2+ subtype
Source: Breast Cancer Res Treat. 2020 Sep 18;185(1):63–72. doi: 10.1007/s10549-020-05925-7 (PMC7500503; doi:10.1007/s10549-020-05925-7)
Supplement: Supplementary file 2 — Supplementary file2 (DOCX 18 kb) [file 10549_2020_5925_MOESM2_ESM.docx]

**Supplementary Table S1** Comparisons of histopathological parameters between NLR/MLR high and low groups among HER2+ patients treated without or with adjuvant trastuzumab

| **HER2+ No adj trastuzumab**  **(n=64)** | **NLR low**  **n (%)** | **NLR high**  **n (%)** | **P**  **value** | **MLR low**  **n (%)** | **MLR high**  **n (%)** | **P**  **value** |
| --- | --- | --- | --- | --- | --- | --- |
| T1 | 22 (58 %) | 12 (46 %) |  | 22 (59.5 %) | 12 (44 %) |  |
| T2-4 | 16 (42 %) | 14 (54 %) | 0.355 | 15 (40.5 %) | 15 (56 %) | 0.235 |
| N0 | 12 (32 %) | 8 (31 %) |  | 13 (35 %) | 7 (26 %) |  |
| N1-3 | 26 (68 %) | 18 (69 %) | 0.945 | 24 (65 %) | 20 (74 %) | 0.432 |
| Grade 1 | 1 (3 %) | 2 (8 %) |  | 1 (3 %) | 2 (7 %) |  |
| Grade 2 | 11 (29 %) | 9 (34 %) |  | 12 (32 %) | 8 (30 %) |  |
| Grade 3 | 26 (68 %) | 15 (58 %) | 0.527 | 24 (65 %) | 17 (63 %) | 0.675 |
| HR Positive | 23 (60.5 %) | 14 (54 %) |  | 24 (65 %) | 13 (48 %) |  |
| HR Negative | 15 (39.5 %) | 12 (46 %) | 0.595 | 13 (35 %) | 14 (52 %) | 0.181 |
| **HER2+ Adj trastuzumab**  **(n=43)** | **NLR low**  **n (%)** | **NLR high**  **n (%)** | **P**  **value** | **MLR low**  **n (%)** | **MLR high**  **n (%)** | **P**  **value** |
| T1 | 11 (39 %) | 12 (80 %) |  | 13 (52 %) | 10 (56 %) |  |
| T2-4 | 17 (61 %) | 3 (20 %) | 0.011* | 12 (48 %) | 8 (44 %) | 0.818 |
| N0 | 10 (36 %) | 5 (33 %) |  | 9 (36 %) | 6 (33 %) |  |
| N1-3 | 18 (64 %) | 10 (67 %) | 0.876 | 16 (64 %) | 12 (67 %) | 0.856 |
| Grade 1 | 0 (0 %) | 1 (6 %) |  | 0 (0 %) | 1 (6 %) |  |
| Grade 2 | 11 (39 %) | 7 (47 %) |  | 10 (40 %) | 8 (44 %) |  |
| Grade 3 | 17 (61 %) | 7 (47 %) | 0.311 | 15 (60 %) | 9 (50 %) | 0.444 |
| HR Positive | 14 (50 %) | 11 (73 %) |  | 14 (56 %) | 11 (61 %) |  |
| HR Negative | 14 (50 %) | 4 (27 %) | 0.139 | 11 (44 %) | 7 (39 %) | 0.738 |

NLR, neutrophil-to-lymphocyte ratio; MLR, monocyte-to-lymphocyte ratio; Adj, adjuvant;

T, tumor classification; N, nodal classification; HR, hormone receptor
